# Nitrate transport in cucumber leaves is an inducible process involving an increase in plasma membrane H^+^-ATPase activity and abundance

**DOI:** 10.1186/1471-2229-12-66

**Published:** 2012-05-09

**Authors:** Miroslav Nikolic, Stefano Cesco, Rossella Monte, Nicola Tomasi, Stefano Gottardi, Anita Zamboni, Roberto Pinton, Zeno Varanini

**Affiliations:** 1 IMSI, University of Belgrade, Kneza Viselslava 1, 11030, Belgrade, Serbia; 2 Faculty of Science and Technology, Free University of Bolzano, 39100, Bolzano, Italy; 3 Department of Agriculture and Environmental Sciences, University of Udine, 33100, Udine, Italy; 4 Department of Biotechnology, University of Verona, 37029, S. Floriano, Italy

## Abstract

**Background:**

The mechanisms by which nitrate is transported into the roots have been characterized both at physiological and molecular levels. It has been demonstrated that nitrate is taken up in an energy-dependent way by a four-component uptake machinery involving high- and low- affinity transport systems. In contrast very little is known about the physiology of nitrate transport towards different plant tissues and in particular at the leaf level.

**Results:**

The mechanism of nitrate uptake in leaves of cucumber (*Cucumis sativus* L. cv. Chinese long) plants was studied and compared with that of the root. Net nitrate uptake by roots of nitrate-depleted cucumber plants proved to be substrate-inducible and biphasic showing a saturable kinetics with a clear linear non saturable component at an anion concentration higher than 2 mM. Nitrate uptake by leaf discs of cucumber plants showed some similarities with that operating in the roots (e.g. electrogenic H^+^ dependence via involvement of proton pump, a certain degree of induction). However, it did not exhibit typical biphasic kinetics and was characterized by a higher *K*_m_ with values out of the range usually recorded in roots of several different plant species. The quantity and activity of plasma membrane (PM) H^+^-ATPase of the vesicles isolated from leaf tissues of nitrate-treated plants for 12 h (peak of nitrate foliar uptake rate) increased with respect to that observed in the vesicles isolated from N-deprived control plants, thus suggesting an involvement of this enzyme in the leaf nitrate uptake process similar to that described in roots. Molecular analyses suggest the involvement of a specific isoform of PM H^+^-ATPase (CsHA1) and NRT2 transporter (CsNRT2) in root nitrate uptake. At the leaf level, nitrate treatment modulated the expression of CsHA2, highlighting a main putative role of this isogene in the process.

**Conclusions:**

Obtained results provide for the first time evidence that a saturable and substrate-inducible nitrate uptake mechanism operates in cucumber leaves. Its activity appears to be related to that of PM H^+^-ATPase activity and in particular to the induction of CsHA2 isoform. However the question about the molecular entity responsible for the transport of nitrate into leaf cells therefore still remains unresolved.

## Background

The availability of nitrate strongly affects both crop productivity and food quality. In fact, in agricultural well-aerated soils, this anion is the main source of nitrogen for the majority of crops. The mechanisms by which nitrate is transported into the roots have been characterized both at physiological and molecular levels. It has been demonstrated that nitrate is taken up by a four-component uptake machinery [1]. Two saturable high-affinity transport systems (HATS) are involved in nitrate transport at low concentration (below 1 mM). One of is constitutive, (cHATS) and the other substrate-inducible (iHATS). The other two systems cLATS and iLATS, the constitutive and inducible low affinity transport systems, respectively, mediate a non-saturable transport, which becomes relevant at concentrations higher than 1 mM. Even at the highest nitrate concentrations likely to be found in the soil solution the transport of the anion into the root cells is an active process coupled to a favourable H^+^ electrochemical gradient created by the plasma membrane (PM) H^+^-ATPase [2,3]. HATS and LATS are encoded by different gene families (*NRT2* and *NRT1*, respectively) whose products act as nH^+^/NO_3_^-^ symporters [4,5]. In *Arabidopsis thaliana* seven *NRT2*[6,7] and eleven *NRT1*[8] gene homologues have been identified. However, only a limited number of them are considered to be responsible for nitrate uptake from the soil [9]. In Arabidopsis NRT2.1 and 2.2 appear to play a major role in iHATS flux and NRT1.1 in iLATS. However, this last gene encodes a dual-affinity nitrate-transporter [10], which might also contribute to iHATS. As far as constitutive transport is concerned, while it has been possible to assign the LATS function to the *AtNRT1.2* gene product, the situation regarding HATS still appears unclear. Regulation of inducible nitrate uptake activity has been shown to take place at transcriptional, post-transcriptional and post-translational levels of *NRT2* genes [10,11].

Once inside root cells, nitrate can be reduced to ammonium by nitrate- and nitrite reductase and then assimilated into organic nitrogen (GS-GOGAT cycle) [12]. Depending on the plant species or when the capacity for nitrate reduction in roots becomes a limiting factor due to high nitrate supply [13], a substantial proportion of nitrate is loaded into xylem vessels and transported upwards to the shoots. Nitrate translocated to the shoots is released from the xylem to the leaf apoplast before being absorbed by the leaf symplast. In contrast to the behaviour of nitrate at root level, little is known about nitrate transport towards different plant tissues Recently, a role in nitrate petiole storage has been attributed to *AtNRT 1.4 9*[14] and the importance of *AtNRT 2.7* for an efficient storage of nitrate in seed vacuoles has been highlighted [15]. Furthermore it has been suggested that the *AtNRT 1.6* gene plays a role in the translocation from maternal tissue to developing embrio [16]. An involvement of *AtNRT1.5* and *AtNRT1.8* genes in root xylem loading and unloading of nitrate, respectively, has also been suggested [17,18]. Notwithstanding the importance of nitrate transport at the level of leaf cells, little or no information is available on this process. It has been demonstrated that bundle sheath cells are sites of intensive net proton excretion, which acidifies the apoplast [19] thus allowing a H^+^/amino acids cotransport across the plasma membrane. From this evidence it has been suggested that the same may hold true for nitrate uptake [13]. However, no information is available on the biochemical and physiological characteristics of this process (e.g. kinetics, inducibility, energy dependence). Furthermore, molecular data reporting the expression of *NRT2*[7,20] and *NRT1*[7,8] genes in leaves, obtained so far, are lacking of functional analyses connecting the pattern of expression to nitrate uptake. Recently, Fan et al. [21] demonstrated that in Arabidopsis leaves the source-to-sink remobilization of nitrate is mediated by *NRT1.7* expression in phloem cells and that its level is related to the source strength of the leaf. In addition, a role of AtNRT1.8 protein in nitrate unloading from xylem tissue of Arabidopsis leaves has been hypothesized [22].

In the present research an attempt was made to characterize the mechanisms of nitrate uptake at the leaf level of N-deprived intact cucumber plants supplied with 4 mM nitrate for up to 24 h. The use of infiltrated leaf disc allowed us to show for the first time that mechanisms operating in leaves possess distinct characteristics as evident from the comparison with the features of root nitrate uptake. To gain information on the regulatory aspect of leaf nitrate uptake and the molecular entities underlying the process we analyzed, during nitrate treatment, the variation of nitrate concentration in different plant compartment (as cell-sap, xylem-sap and apoplastic fluid) and the behavior of *NRT* genes and PM H^+^-ATPase.

## Results

**Figure 1:**
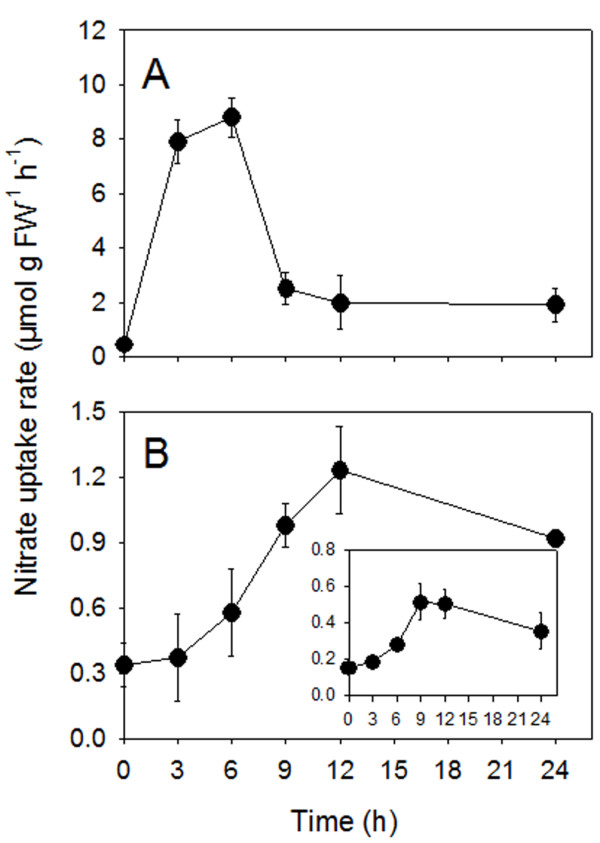
**Net nitrate-uptake rate by cucumber roots (A) and leaf discs (B).** Cucumber plants were grown in N-free nutrient solution for 5 days before being exposed to 4 mM nitrate. Uptake by roots was measured at 0.2 mM nitrate, while by leaves either at 0.2 (inside) or 2 mM nitrate. Data are means ± SD of three experiments with four replicates.

Figure 1 shows the pattern of high affinity nitrate uptake (measured at 0.2 mM) by roots and leaf discs of N-deprived cucumber plants exposed to 4 mM nitrate up to 24 h. As shown in Figure 1A, when the plants were exposed to a nutrient solution containing nitrate, at the root level the net nitrate-uptake rate rapidly increased reaching the maximum level after 3 to 6 h of exposure with the solution (induction). At this time, the magnitude of the net nitrate-uptake capacity was 8-fold higher than that recorded in roots at the beginning of the experiment. Thereafter, a decline in net nitrate-uptake rate was observed. When the nitrate-uptake was measured in leaf discs (Figure 1B), an enhancement in nitrate-uptake rate was evident and became maximal after 9 to 12 h of treatment. In this tissue, the extent of the induction was of about 2 fold. Moreover, prolonging the experiment up to 24 h, the rate of net nitrate uptake only slightly decreased. The same pattern, but with higher extent of induction (4 fold), was also observed when the leaf discs were put in the contact with an uptake solution with higher (2 mM) nitrate concentration (Figure 1B, inside). Thereafter this concentration was used for all the other leaf-uptake experiments.

**Figure 2:**
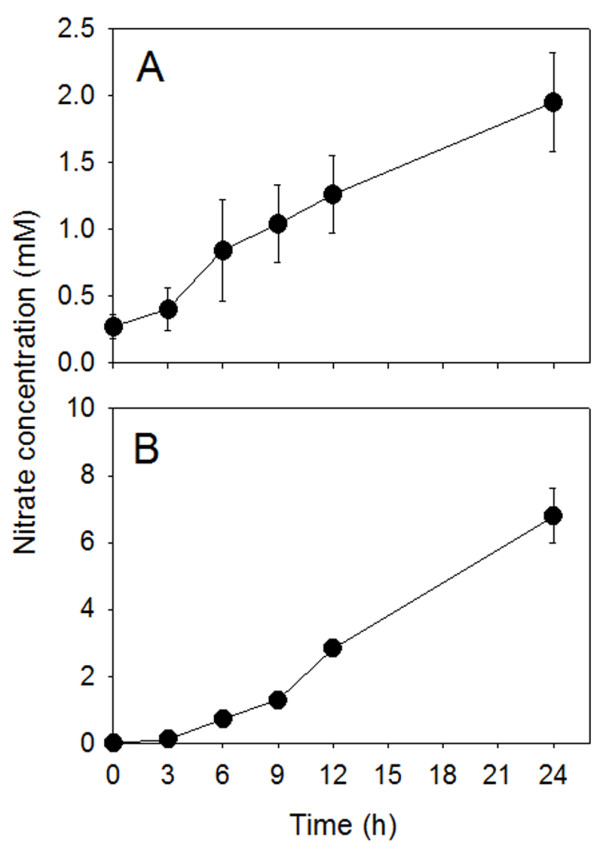
**Nitrate concentration in the cell sap of cucumber roots (A) and leaves (B).** Cucumber plants were grown in N-free nutrient solution for 5 days before being exposed to 4 mM nitrate. Cell saps were collected as described in Methods. Data are means ± SD of three experiments with four replicates. For most of the points, SD values are very low and masked by dot symbols.

Cell-sap analysis showed that supply of nitrate to the N-deprived plants caused a progressive increase in nitrate concentration either in roots or leaves (Figure 2); in these latter, the magnitude of anion concentration was about 4-fold higher than that measured at the root level after 24-h treatment.

**Figure 3:**
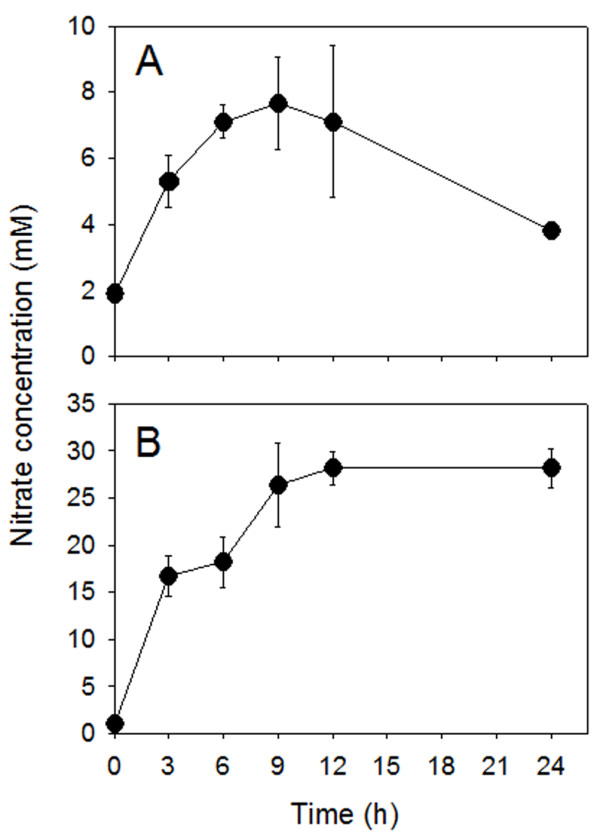
**Nitrate concentration in the leaf apoplastic fluid (A) and xylem sap (B) of cucumber.** The concentration of nitrate in the leaf apoplastic fluid was calculated due to multiplication of the nitrate concentration measured in the apoplastic washing fluid (AWF) by the dilution factor of 6 (see Methods). Treatments of the plants and statistics as reported in the legend of Figure 1.

In xylem sap, nitrate concentration (Figure 3B) rapidly increased up to 12 h after the anion supply and thereafter remained constant up to the end of the experiment (24 h). In the leaf-apoplastic fluid, anion concentration increased as a consequence of nitrate supply to the plants, being maximal after 9 h of treatment (Figure 3A); afterwards, the levels of nitrate concentration in this fluid decreased rapidly.

**Figure 4:**
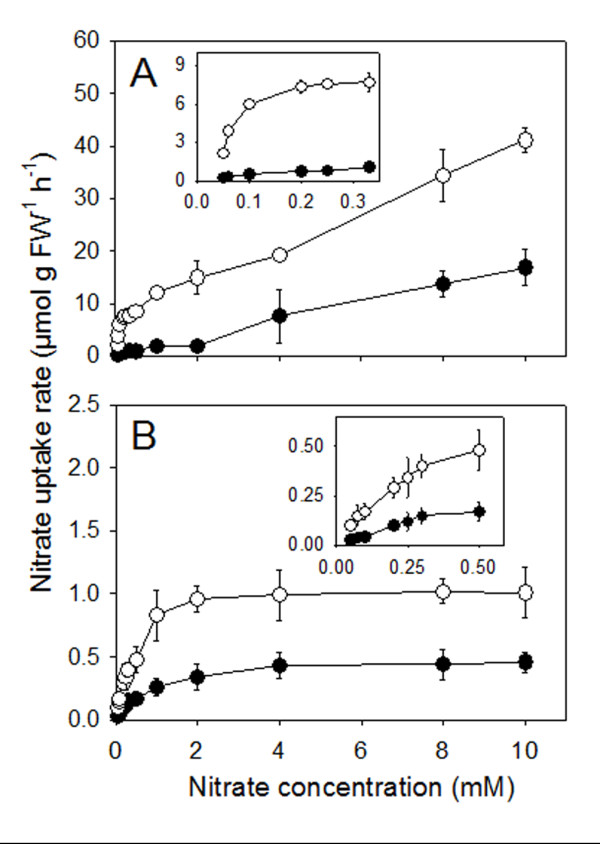
**Substrate-concentration effect on the net nitrate uptake by intact roots (A) and leaf discs (B).** Full circles, before nitrate supply; open circles, 4- (roots) or 12-h (leaves) after supply of 4 mM nitrate. The variable concentrations of potassium in the uptake solutions (0.05-10 mM KNO_3_) were compensated with K_2_SO_4_. Data are means ± SD of two experiments with four replicates.

In Figure 4 are reported the kinetic curves of net nitrate uptake by roots and leaf discs measured respectively after 4 h and 12 h of exposure of N-deprived plants to 4 mM nitrate; as control, tissues collected from N-deprived plants before starting the treatment, were used. At root level of nitrate-induced plants, the nitrate uptake rate increased with the rise of the external anion concentration, showing a biphasic pattern with a typical saturation profile up to 0.33 mM nitrate and a linear kinetic at higher anion concentrations (Figure 4A). By contrast, net nitrate uptake by leaf discs of nitrate-induced plants displayed only saturation kinetics (Figure 4B). Similar patterns but with lower rates of uptake were observed in both tissues of the control (non induced) plants.

The kinetic parameters were calculated by using the Lineweaver-Burk double reciprocal method, in the low external nitrate-concentration range, where the curves displayed saturation kinetics in both roots and leaves. Results reported in Table 1 show that, when the plants were exposed to a nutrient solution containing nitrate, *V*_max_ significantly increased either at root (+464%) or leaf (+133%) level. On the other hand, the apparent *K*_m_ values decreased as a consequence of the nitrate treatment, this behaviour being particularly evident at the root level.

**Table 1:** Kinetic parameters for net-nitrate uptake by roots and leaves

Parameters*	**Roots**		**Leaves**	
	**N-free**	**+Nitrate for 4 h**	**N-free**	**+Nitrate for 12 h**
*K*_m_ (mM)	0.35	0.12	0.79	0.52
*V*_max_ (μmol NO_3_^-^ g FW^-1^ h^-1^)	2.04	11.52	0.48	1.12

^*^Calculated in the 0.05-0.5 mM concentration range. Values for *K*_m_ and *V*_max_ were obtained using the Lineweaver-Burk method (double reciprocal method) of data fitted to the Michaelis-Menten equation.

**Figure 5:**
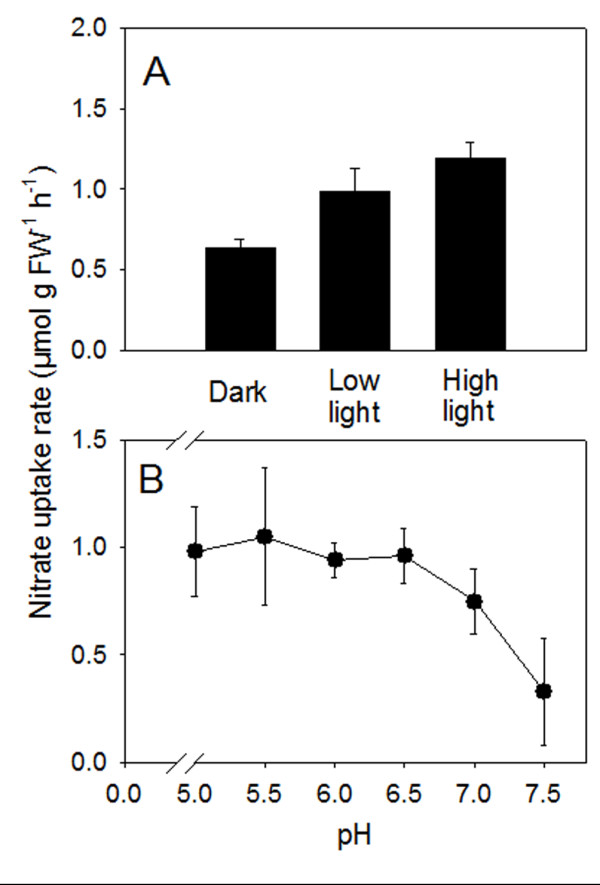
**Light- (A) and pH-dependency (B) of the net nitrate-uptake by leaf discs.** N-deprived cucumber plants were treated with 4 mM nitrate for 12 h. Plants were exposed to dark, low (100 μmol m^-2^ h^-1^) or high (500 μmol m^-2^ h^-1^) light for 8 h before collecting leaf discs. The pH dependency was evaluated using 10 mM MES-NaOH buffer (pH 5.0, 5.5, 6.0 or 6.5) or 10 mM Hepes-NaOH (pH 7.5). Data are means ± SD of three experiments with four replicates.

Net nitrate uptake by leaf discs displayed a marked dependence upon the light, greatest values being reached when the assays were performed in the presence of high light intensity (500 μmol m^-2^ h^-1^; Figure 5A).

**Figure 6:**
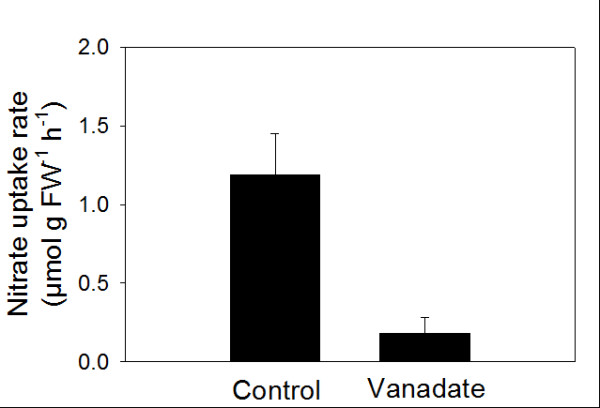
**Effect of PM H**^**+**^** −ATPase inhibitor vanadate on the net nitrate uptake by leaf discs.** N-deprived cucumber plants were subjected to 4 mM NO_3_^-^ for 12 h. Sodium vanadate was applied in the uptake solution at a final concentration of 500 μM. After addition of vanadate the pH of the uptake solution was re-adjusted to 6.0 with 0.1 mM HCl. Data are means ± SD of four replicates.

In order to evaluate the dependence of nitrate uptake by leaf discs on the activity of the PM H^+^-ATPase, experiments were also performed in the presence of 500 μM vanadate in the uptake medium. The presence of the inhibitor of the proton pump in the uptake medium drastically lowered (−84%) the capability of the leaf discs to take up nitrate from the external solution (Figure 6). The dependence of net nitrate uptake by leaf discs upon the pH of the external medium was evaluated at pH intervals ranging from 5.0 to 7.5. Net nitrate-uptake rates were essentially unaffected by pH raising from 5.0 up to values of 6.5, while a progressive inhibition was observed when the solution was buffered at higher pH (Figure 5B).

**Figure 7:**
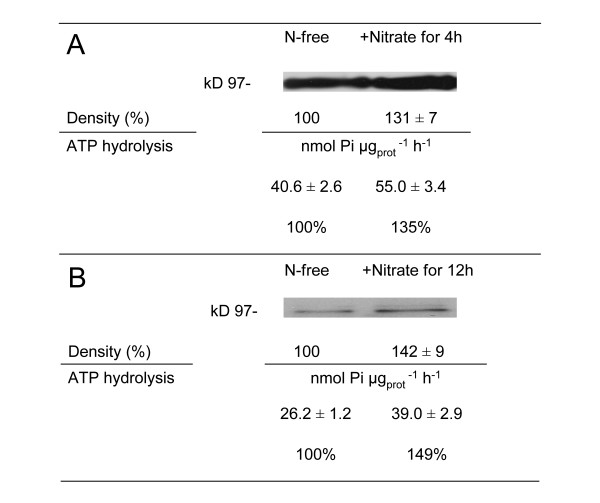
**Hydrolytic-activity and western-blot analyses of PM H**^**+**^**-ATPase from cucumber roots (A) or leaves (B).** The plants were treated as described in the legend of Figure 4. Data are means ± SD of three independent experiments with three replicates. Immunodetection of the enzyme isolated from the three independent experiments was performed using antibodies raised against the C-terminal part of the Arabidopsis AHA3 PM H^+^-ATPase. Blots of a representative experiment are shown.

To verify whether the modification of PM H^+^-ATPase occurred concomitantly with the nitrate-uptake induction, PM -enriched vesicles were isolated from roots and leaves of N-deprived or nitrate-induced (roots after 4 h and leaves after 12 h of exposure to 4 mM nitrate, respectively) of intact cucumber plants. Based on the effects of selective inhibitors on PM H^+^-ATPase activity, membrane preparations appeared enriched in plasma membrane vesicles. Vanadate inhibited PM H^+^-ATPase activity by 85% and 89% in leaf and root respectively. Moreover, the nitrate treatment did not significantly modify the composition of the isolated membrane-vesicles preparations. The hydrolytic activities and the amounts of PM H^+^-ATPase measured in the vesicle preparations are presented in Figure 7. When plants were placed in contact with nitrate for 4 h the specific activity of root PM H^+^-ATPase increased (+35%) in relation to that measured in membrane vesicles isolated from roots of N-deprived plants (Figure 7A). Western blot analysis indicated a relative increase (+31%) of the enzyme-steady-state level within plasma-membrane proteins from nitrate-induced roots. For the leaves, following treatment with nitrate for 12 h, the hydrolytic activity and the relative amount of PM H^+^-ATPase were respectively (+50%) and (+42%) higher than the comparative values detected in vesicle preparations isolated from leaves of N-deprived plants (Figure 7B).

**Figure 8:**
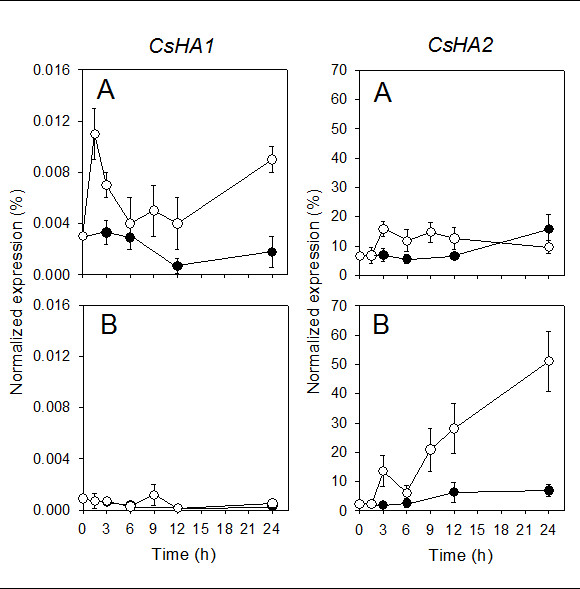
**Expression analyses of genes coding for PM H**^**+**^**-ATPase in cucumber roots (A) and leaves (B).** The plants were treated as described in the legend of Figure 4. Relative expression of *HA1* and *HA2* genes coding for PM H^+^-ATPase was analyzed by real-time RT-PCR. Data are means ± SD of three independent experiments run in triplicate. Changes in gene expression were calculated on the basis of expression levels of *alpha-tubulin* gene.

Transcriptional activity of PM H^+^-ATPase genes was investigated by real-time RT-PCR analyses using primers designed from gene sequences of two different H^+^-ATPase isoforms known to be present in cucumber plants: *CsHA1* (AJ703810) and *CsHA2* gene (AJ703811). PM H^+^-ATPase genes showed different expression patterns during the treatment. At root level (Figure 8A), two distinct peaks in transcript abundance were observed for *CsHA1* after 1.5 h and 24 h of nitrate exposure; expression of *CsHA2* whose transcript levels were higher, was less modified by the treatment. When the abundance of transcripts was analyzed at the leaf level (Figure 8B), results show that the expression level of *CsHA1* was unaffected by the nitrate supply to the plants while the expression of *CsHA2* increased progressively after nitrate supply to intact plants up to the end of the treatment.

**Figure 9:**
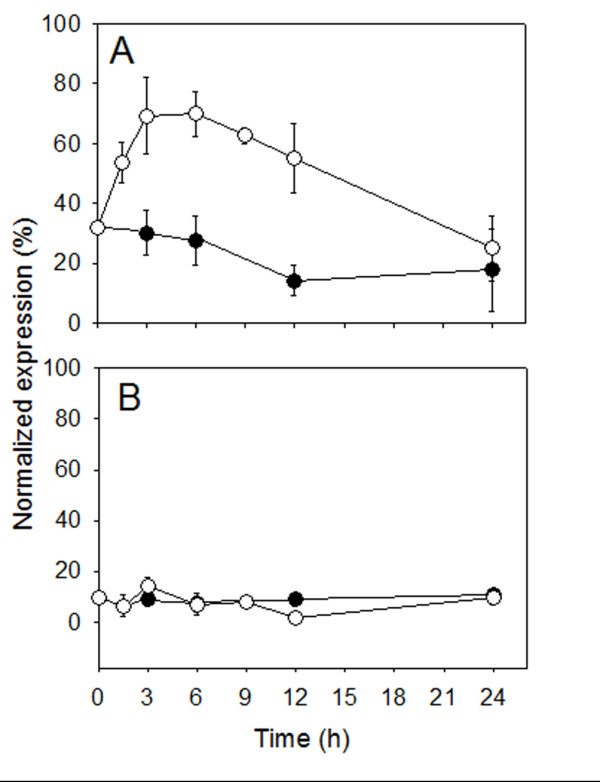
**Expression analyses of genes coding for NRT2 in cucumber roots (A) and leaves (B).** The plants were treated as described in the legend of Figure 4. Black circles, control (N-deprived plants without nitrate treatment); white circles, N-deprived plants treated with 4 mM nitrate. Relative expression of *NRT2* gene was analyzed by real-time RT-PCR. Data processing reported in the legend of Figure 8.

Expression of the high-affinity nitrate transporter (*CsNRT2*) gene at the root level increased during the first 3 h of treatment; after 6 h the levels of transcript abundance progressively fell reaching a value similar to that of untreated plants (Figure 9A). At the leaf tissue, expression level of this gene was unaffected by the nitrate treatment of plants (Figure 9B). From the findings of these transcriptional profiles which suggest that other molecular mechanisms may be involved in nitrate transport in leaf, we performed experiments aimed at identifying genes putatively involved in the transport of nitrate in leaf. Degenerate primes were used to amplify sequence of putative members of the *NRT2* and *NRT1* gene families using cDNA samples obtained from the total RNA extracted from both roots (sampled at 1 h and 30 min and 3 h after the treatment) and leaf tissues (sampled at 9 h after the treatment). Only with one combination of primers (NRT2forA and NRT2rev) a PCR amplification fragment of about 200 bp was obtained. For each cDNA sample, the amplified cDNA fragments were cloned and two different clones were sequenced. The analysis of the obtained sequences (Blastn against the NCBI nucleotide database) revealed that all root cDNA sequences (at 1 h and 30 min and at 3 h after treatment) had a 100% sequence identity with the same *Cucumis sativus* high-affinity nitrate transporter *CsNRT2* mRNA (AY584189), whose transcriptional levels were previously analyzed by Real-time RT-PCR (Figure 9). On the other hand, no specific amplified fragments resulted from the leaf tissues.

The same cDNA samples were used as templates for reactions with degenerate primers to amplify two sequence regions of the *NRT1* gene family. Only the NRT1forA and NRT1revA combination of primers amplified a fragment of about 100 bp. However, the blastn analysis of the sequences obtained from two clones for each cDNA samples showed no similarity to known nucleotide sequence encoding NRT1 proteins.

## Discussion

In many plant species, considerable amount of nitrate taken up by roots is transported to the leaf cells, where it undergoes reduction to ammonium and conversion to amino acids and other nitrogen containing molecules. Consequently, nitrate unloaded from the xylem must be taken up into leaf cells. The mechanism of nitrate uptake across the root plasma membrane of higher plants is suggested to be a H^+^/NO_3_^-^ symport most likely with a 2:1 stoichiometry [23,24]. The accumulated evidence from kinetic studies indicates that roots possess different nitrate uptake systems that have been defined according to their response to external nitrate in terms of concentration-dependence (high- and low-affinity) and inducibility (constitutive and inducible) for review see e.g. [1,25-27]. The time course experiments of nitrate uptake by roots of nitrate-depleted cucumber plants confirmed a typical response [28]: net uptake rates rapidly and consistently increased upon roots exposure to the anion reaching a maximum 3 to 6 h (Figure 1A) after the beginning of the treatment. Thereafter a steep decline of the absorption rate occurred. This pattern, together with saturable kinetics (0.05-0.40 mM range), clearly indicates the occurrence in cucumber roots of iHATS during nitrate induction period. Furthermore, at the nitrate concentration higher than 2 mM linear non saturable kinetics was evident, thus indicating that the features of nitrate uptake in root of cucumber plants are similar to those already described in several plant species [29].

Once taken up by roots, nitrate is loaded into the xylem vessel and translocated to the shoots. In general, the root nitrate reductase activity is usually lower in the region where the nitrate uptake is higher and is responsible for nitrate translocation from these root parts to the shoots [30]. While the uptake of nitrate into the root symplast depends on the concentration in the outer medium, the xylem loading process depends mainly on the shoot demand and the content of reduced N-compounds in the phloem [31]. In the present study, nitrate concentration in the xylem sap rapidly increased following anion supply (3–9 h of induction period), resulting in a higher leaf apoplastic concentration (Figure 3). This implies that leaf cells also experience fluctuating concentration of nitrate in their apoplast. However, as yet information concerning the physiological features of nitrate uptake in leaf tissues is lacking in the literature. Our data, obtained with leaf discs, demonstrate that as in the case of roots, the uptake of nitrate in leaves appears to be an inducible process; the enhancement in nitrate-uptake rate reached the maximum after 9 to 12 h of the anion treatment (Figure 1B). However, the intensity of induction and the entity of the decline of uptake rate were lower compared to the root, possibly indicating that uptake mechanisms operating in leaf tissues possess different sensitivity to regulatory effectors. Net nitrate uptake by the leaf discs was stimulated by light (Figure 5A) which is in accordance with the findings of Peuke and Jeschke [32] obtained with barley roots.

In contrast to the roots, net nitrate uptake by leaf discs of nitrate-induced cucumber plants did not exhibit typical biphasic kinetics (Figure 4B). Although foliar nitrate uptake shows some similarities with the root process (e.g. electrogenic H^+^ dependence via involvement of proton pump, a certain degree of induction), the kinetic pattern (saturable and higher *K*_m_ values in leaves) and the intensity of induction indicate that the mechanism(s) operating at the leaf level might be different from those present in roots, which is usually attributed to *NRT2.1,* and *NRT1.1* genes. Furthermore, *K*_m_ values obtained for cucumber leaf tissues (Table 1) are out of the range (0.007-0.110 mM) recorded in roots of several different plant species [33] suggesting an adaptation to higher apoplastic nitrate concentration and the involvement of transporters different from those responsible for nitrate uptake at the root soil interface [9]. Recently, kinetics similar to those shown in Figure 4B (saturation at about 5 mM nitrate) have been demonstrated for the low-affinity nitrate transporter NRT1.7 in the minor vein phloem of Arabidopsis leaves (measured as currents in *NRT1.7*-injected oocytes) [21].

The involvement of PM H^+^-ATPase in nitrate uptake has been demonstrated in maize roots exposed to fluctuating external nitrate concentration [2,3]. Data presented here confirm that also in cucumber roots higher level of nitrate uptake rates are related to an increase in PM H^+^-ATPase activity and quantity (Figure 7A). Furthermore, nitrate treated roots exhibited differential regulation at transcriptional level of two plasma membrane proton pump isoforms (Figure 8A). These data confirm that a different isogene of PM H^+^-ATPase could play a different role in mineral nutrition as suggested for nitrate in maize [3] and iron nutrition in cucumber [34,35] as well as for phosphorus acquisition by white lupin [36]. Data obtained using isolated plasma membrane vesicles from leaves of cucumber plants treated for 12 h with nitrate (peak of nitrate foliar uptake rate) show, for the first time, that also in this tissue the activity and quantity of the plasma membrane proton pump increase (Figure 7B) This finding suggests an involvement of the enzyme in the nitrate uptake process in leaves similar to that described in roots. The transcriptional analysis of *CsHA1* and *CsHA2* in leaves highlighted a main putative role of the latter isogene in the process (Figure 8B). Interestingly the expression of this gene was observed in leaves of the cucumber plant irrespective of iron nutritional status this behaviour being different from that of *CsHA1* whose expression is modulated in the root both in response to iron shortage [34] and the presence of nitrate (present work).

Data obtained from Real-Time RT-PCR experiments, aimed at analyzing the involvement of the only high affinity nitrate transporter (*CsNRT2*) so far known in cucumber, clearly showed that this gene plays a role in nitrate uptake in roots (Figure 9A). However this gene was not modulated in leaves (Figure 9B), suggesting that other molecular mechanisms underlie the phenomenon of nitrate uptake in this tissue. The experiments performed to identify other isogenes of high affinity nitrate transporters using degenerate primers (see Methods) indicate evidence of expression of only *CsNRT2* in root tissue at any time of the nitrate treatment. On the other hand the same approach did not allow amplification of the sequence portion of *NRT2* genes in leaves. It has been recently hypothesized that AtNRT1.8, nitrate responsive in roots and expressed in xylem tissues both in roots and leaves, is involved in xylem unloading [18]. A product of this gene may play a role, at least in part, in the process of nitrate uptake in foliar tissues [22]. Bearing this in mind we used degenerate primers to attempt identify the putative low affinity nitrate transporter (NRT1). Our finding of lack of evidence of the *NRT1* gene either in roots or leaves does not necessarily imply that these genes are not expressed in leaf tissues. This is because of the very low sequence similarity among the members of this gene family [8]. The molecular entity responsible for the transport of nitrate into leaf cells therefore still remains unresolved.

## Conclusions

Our data provide for the first time evidence that a saturable and substrate-inducible nitrate uptake mechanism operates in cucumber leaves. Its activity appears to be related to that of PM H^+^-ATPase activity and in particular to the induction of CsHA2 isoform.

## Methods

### Plant material and growth conditions

**Figure 10:**
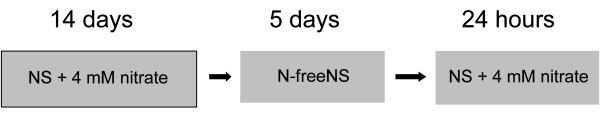
**Schematic representation of the plant-growth conditions.** NS, nutrient solution.

Cucumber (*Cucumis sativus* L. cv. Chinese long) was grown under controlled environmental conditions in a growth chamber with light/dark regime of 16/8 h, temperature regime of 24/20°C, photon flux density of approximately 300 μmol^-2^ s^-1^ at plant height and relative humidity of about 70%. After soaking in 1 mM CaSO_4_ overnight, seeds were germinated on filter paper moistened with 1 mM CaSO_4_ solution in darkness for 5 d. The 5-d-old seedlings were then transferred (4 plants per 2-L plastic pots) to a full-strength nutrient solution containing (mM): 2 Ca(NO_3_)_2_, 0.7 K_2_SO_4_, 0.1 KH_2_PO_4_, 0.1 KCl, 0.5 MgSO_4_, and (μM): 10 H_3_BO_3_, 0.5 MnSO_4_, 0.2 CuSO_4_, 0.1 ZnSO_4_, 0.01 (NH_4_)_6_Mo_7_O_24_, 80 Fe^III^-EDTA. The nutrient solution was renewed every 2 d and continuously aerated. Cucumber plants were grown for 14 days in full-strength nutrient solution and then transferred for 5 days to a N-free nutrient solution, in order to promote a de-induction of root nitrate uptake mechanism. Thereafter nitrate as Ca(NO_3_)_2_ was added to the nutrient solution at a concentration of 4 mM N up to 24 h. The plant growth conditions are illustrated in Figure 10.

### Determination of nitrate

Nitrate in different samples (i.e. uptake solution, xylem sap and tissues) was determined according to the slightly modified method of Cataldo et al. [37]. Aliquots of 0.1 mL were mixed thoroughly with 0.4 mL of 5% (w/v) salicylic acid in concentrated H_2_SO_4_. After 20 min incubation at room temperature, 9.5 mL of 2 M NaOH were added. The samples were cooled to room temperature and nitrate concentration determined spectrophotometrically by measuring the absorbance at 410 nm.

### Measurement of net nitrate uptake by intact roots and leaf discs

After different periods of exposure to 4 mM nitrate (0, 3, 6, 9, 12, 24 h, respectively), roots of intact cucumber plants were rinsed shortly in 1 mM CaSO_4_ and subsequently immersed in 40 mL uptake solution containing 0.2 mM KNO_3_, 0.5 mM CaSO_4_ and 10 mM 2-(N-morpholino) ethanesulfonic acid (MES)/NaOH (pH 6.0) at 25°C. Nitrate depletion from the solution was measured over 10 min by removing an aliquot of 0.1 mL every 2 min for determination of nitrate. The nitrate uptake rate was calculated by linear regression analysis according to Pinton et al. [38].

At the same time intervals, the fully expanded leaves were excised and major veins were removed. Leaf discs of 1 cm diameter (20 discs per sample) were taken and after short washing in 0.5 mM CaSO_4_, were vacuum-infiltrated in 5 mL solution containing 1 mM CaSO_4_ and 5 mM MES/NaOH (pH 6.0) to ensure equilibrium between apoplastic and external solutions. Thereafter, the washing solution was replaced with 5 mL uptake solution containing 2 mM KNO_3_, 0.5 mM CaSO_4_ and 5 mM MES/NaOH (pH 6.0) at 25°C. Uptake was carried out under light (500 μmol photons m^-2^ s^-1^) with continuous aeration of the solution. Depletion of nitrate from the solution was monitored over 15 min as described above.

### Cell sap preparation and collection of xylem sap

At the end of the experiments, excised roots and leaves were immediately frozen in liquid nitrogen and stored at −80°C. Cell sap samples were prepared according to Nikolic et al. [39] by thawing the defrosted tissues followed by centrifugation at 10,000 *g* for 15 min.

Xylem sap was obtained by exudation after cucumber plants were decapitated at the stem ∼ 2 cm above the root base [38]. Silicon tubes were fixed over decapitated stem and xylem sap was collected for 2 h after discarding the exudates obtained during the first few minutes.

### Collection of the leaf apoplastic washing fluid (AWF)

Leaflets devoid of a main midrib were infiltrated with ice-cold deionised water buffered at pH 5.5 (5 mM MES/NaOH) in a vacuum-desiccator in several cycles of reducing the pressure, and left to slowly revert to atmospheric pressure until leaflets become completely dark, carefully blotted dry, rolled over a plastic tube, orientated with the cut edge up and fixed into a plastic 25-mL syringe. The leaves-filled syringes were then centrifuged at 1,500 *g* for 15 min at 4°C. In all samples, symplastic contamination detected as a malate dehydrogenase (E.C. 1.1.1.37; a mitochondria marker enzyme) activity was below 0.5% of the activity measured in the total leaf homogenate (not shown).

### Determination of apoplastic air and water volumes

Apoplastic air and water volumes of cucumber leaf were determined by leaf infiltration with silicone oil and indigo carmine, respectively, according to Husted and Schjoerring [40]. The apoplastic water volume was about 0.07 cm^3^ H_2_O cm^-3^ tissue, and the apoplastic air volume was about 0.36 cm^3^ air cm^-3^ tissue. The concentration of nitrate in the AWF was thus corrected by multiplication with the dilution factor of 6, which corresponds to the nitrate concentration in the leaf apoplastic fluid.

### Isolation of plasma membrane (PM) vesicles

PM vesicles were isolated from leaf samples as described in Tomasi et al. [36] with slight modifications. Briefly, 5 g of leaves were vacuum-infiltrated with a 20 mL of ice-cold extraction medium containing: 250 mM sucrose, 2 mM MgSO4, 2 mM ATP, 10% (v/v) glycerol, 10 mM glycerol-1-phosphate, 0.16% (w/v) bovine serum albumin, 2 mM ethylene glycol tetraacetic acid, 2 mM DTT, 5.7% (w/v) choline-iodide, 1 mM phenylmethylsulfonyl fluoride, 20 μg ml^–1^ chymostatin, 10 nM okadeic acid, 25 mM MES-1,3-bis[tris(hydroxymethyl)-methyloamino] (BTP) pH 7.6 and 0.5 g g^–1^ FW polyvinylpolypyrrolidone, in order to ensure an equilibrium between apoplastic and external solutions, and then homogenized with a mortar and pestle. The homogenates were filtered through four layers of cheesecloth and the suspensions were subjected to differential centrifugation steps at 2°C: 1,500 g for 5 min (pellets discarded); 9,800 g for 20 min (pellets discarded); 83’400 g for 30 min (pellets recovered); and 83,400 g for a further 30 min. Microsomes, gently resuspended in 1.2 mL of homogenization medium (without PVPP), were loaded onto a discontinuous sucrose gradient made by layering 2 mL of sucrose solution (1.13 g cm-3) onto a 3 mL sucrose (1.17 g cm-3) cushion, and centrifuged at 107,600 g for 2 h. The sucrose solutions were prepared in 5 mM MES-BTP pH 7.4, and contained all of the protectants present in the homogenization medium. Vesicles migrating to the 1.13/1.17 g cm^-3^ interface were collected, diluted with homogenization medium, and centrifuged at 122,400 g for 30 min. The pellets were resuspended in a medium containing 250 mM sucrose, 10% (v/v) glycerol, 1 mM dithiothreitol, 50 μg mL^–1^ chymostatin, 10 nM okadeic acid and 2 mM MES-BTP pH 7.0, were immediately frozen in liquid nitrogen, and stored at −80°C until use.

### Measurement of PM H^+^-ATPase activity and membrane protein content

PM H^+^-ATPase activity was measured at 38°C in a 0.6 mL reaction [50 mM MES-BTP pH 6.5 or pH 6.2 to 8.0 (for the pH dependency assay), 5 mM MgSO4, 100 mM KNO3, 600 μM Na2MoO4, 1.5 mM NaN3, 5 mM ATP-BTP (pH 6.5), 0.01% (w/v) Brij 58 (polyoxyethylene 20 cetyl ether), plus or minus 100 μM V_2_O_5_; the vanadate-dependent activity was 85 ± 3% in the leaf samples and 89 ± 4% in the root samples]. The reaction was started by addition of membrane vesicles containing 0.5 μg of total protein; after 30 min, the reaction was stopped and colour developed as previously described by Santi et al. [2]. Inorganic phosphate was quantified spectrophotometrically at 705 nm as described by Forbush [41]. Protein content was determined according to Bradford [42], using BSA as standard, after solubilizing membrane vesicles with 0.5 M NaOH [43].

### Western blots

Equal amounts of protein isolated at the different time points were loaded, electrophoresed in an 8% w/v SDS-PAGE gel and transferred to a Protran BA 83 nylon membrane (0.2 μm, Biorad, Hercules, USA) with a semi-dry transfer system (Trans-blot SD, Biorad, Hercules, USA). For leaf and root samples were loaded 30 and 15 μg protein respectively. For the PM H^+^-ATPase blot, a polyclonal antibody against the C-terminal part of the *Arabidopsis thaliana* AHA3 plasma-membrane H^+^ −ATPase. Homogeneity of the loading and transfer was checked using Ponceau red staining on the membrane. Secondary antibodies (Goat anti-rabbit IgG alkaline phosphatase conjugate, Biorad, Hercules, USA) were used and the immunodetection was performed using the standard BCIP/NBT protocol (Promega, Madison, USA). Relative-intensity band quantifications were determined using ImageJ (1.40 g; http://rsb.info.nih.gov/ij/).

### Gene expression analysis

At the harvesting times, samples of roots and whole leaves were collected, immediately frozen in liquid nitrogen and conserved until further processing at −80°C. RNA extractions were performed using Invisorb Spin Plant RNA kit (Invitek, Berlin, Germany) following manufacturer’s instructions. 1 μg of total RNA (checked for quality and quantity using a spectrophotometer, followed by a migration in an agarose gel) of each sample was retrotranscribed using 1 pmol of Oligo d(T)_23_VN (Sigma-Aldrich, Milan, Italy), 15 U Prime RNase Inhibitor (Eppendorf, Hamburg, Germany) and 10 U M-MulV RNase H^-^ for 1 h at 42°C (Finnzymes, Helsinki, Finland) following the manufacturer’s instruction. After RNA digestion with 1U RNase A (USB, Cleveland, USA) for 1 h at 37°C, gene expression analyzes were performed by adding 0.1 μL of the cDNA to FluoCycleTM sybr green (20 μl final volume; Euroclone, Pero, Italy) in a DNA Engine Opticon Real-Time PCR Detection (Biorad, Hercules, USA).

Primers used (Tm = 58°C) were the following: for *HA1* gene (AJ703810), 5’-CGCCTTTACGACCAAGAAAG-3’ and 5’-CTGGTTGGAGGCCATGTAAG-3’, for *HA2* gene (AJ703811), 5’-GCGACCTGGACTTCTATTGG-3’ and 5’-TCCGATTCCCTTGATCTTTG-3’, for *Nrt2* gene (AY584189), 5’-CAATAGGAGCACAAGCAGCA-3’ and 5’-TCCAAAGTTTCCACCAGCTC-3’, and as housekeeping gene (*alpha-tubulin*; AJ715498), 5’-GGAACACACTGACGTTGCTG-3’ and 5’-CCTGGGATACAAGACGGTTG-3’). Triplicates were performed on three independent experiments; analyses of real-time result were performed using Opticon Monitor 2 software (Biorad, Hercules, USA) and R (version 2.8.0; http://www.r-project.org/) with the qPCR package (version 1.1-7; http://www.dr-spiess.de/qpcR.html). Efficiencies of amplification were calculated following the authors’ indications [44]: PCR efficiencies were 75.3, 94.3, 77.25 and 87.6% for *HA1, HA2, Nrt2* and *TUA* genes, respectively. Computation of the graphical representation and statistical validation (*t*-test) were performed using SigmaPlot 11.0 (Systat Software, Point Richmond, USA), considering the differences in the PCR efficiencies and indicating the relative expression of the gene of interest versus the expression of *alpha-tubulin* gene in the considered sample.

### Cloning of putative members of *NRT1* and *NRT2* gene families

In order to clone putative members of NRT1 and NRT2 gene families RNA samples obtained as previously described from root and leaf tissues were used. For each sample, after the removing of DNA traces with RQ1 RNase-free DNase (Promega, Madison, USA) treatment, 1 μg of total RNA (checked for quality and quantity using a spectrophotometer, followed by a migration in an agarose gel) was retrotranscribed using 1 pmol of Oligo d(T)_23_VN (Sigma Aldrich, Milan, Italy), 15 U Prime RNase Inhibitor (Eppendorf, Hamburg, Germany) and 10 U M-MulV RNase H^-^ for 1 h at 42°C (Finnzymes, Helsinki, Finland) following the manufacturer’s instruction. A RNA digestion with 1U RNase A (USB, Cleveland, USA) was finally performed for 1 h at 37°C. One micro liter of the obtained cDNA samples was used as template in PCR reaction performed with different pairs of degenerate primers in order to amplify a sequence portion of *NRT2* and *NRT1* gene families respectively. The degenerate primers were designed using CODEHOP [45] on the basis of a multiple sequence alignment. The NRT2 sequence alignment was obtained using the coding sequences (CDS) of the following mRNAs: *AtNRT2.1* (NM100684), *AtNRT2.2* (NM100685), *AtNRT2.4* (NM125470), *AtNRT2.7* (NM121461), *CsNRT2.1* (AY584189), *HvBCH1* (U34198), *LeNRT2.1* (AF092655), *NtNRT2.1* (AJ557583), *PpNRT2.1* (AB097402), *TaNRT2.1* (AF332214) and *ZmNRT2.1* (NM001111725), while the NRT1 sequence alignment using the CDSs of the following mRNAs: *AtNRT1.1* (NM101083), *AtNRT1.5* (NM102980), *OsNRT1* (AF140606), *TaNRT1.1* (AY587265) and two cucumber unigenes (CU11446 and CU12956) of the Cucurbit Genomics Database [46]. The PCR reaction for the amplification of NRT2 gene family sequence was performed using the NRT2forA (5’-ACCACHGAYAYYGNTTYGC-3’) and NRT2forB (5’-AAYTAYMGDACNTTGATYTT-3’) in combination with the same reverse primer NRT2rev (5’-CCDGCRGTTTGNARMATCCA-3’). The amplification condition was optimized using the following cycling conditions: 94°C for 5 min, followed by 5 cycles of 94°C for 45 s, 46°C for 45 s with an 1°C increase at each cycle, 72°C for 30 s and 30 cycles of 94°C for 45 s, 51°C for 45 s, with a final step at 72°C for 5 min. The PCR reaction for the amplification of NRT1 gene family sequence was performed using two combination of primers, NRT1forA (5’-CGTCGCCACCTTCGGCDSNGAYCARTT-3’) with NRT1revA (5’-GGTGACCATGGCNGCNYKRTC-3’) and NRT1forB (5’-CGACCAGTTGAGGTTGTTCGAYMRNGCGC-3’) with NRT1revB (5’-TCATCCGGATGATGCACTTNAVYTCYTC-3’) using the following cycling conditions: 94°C for 5 min, followed by 5 cycles of 94°C for 45 s, 50°C for 45 s with an 1°C increase at each cycle, 72°C for 30 s and 30 cycles of 94°C for 45 s, 55°C for 45 s, with a final step at 72°C for 5 min. The obtained PCR products were inserted into a pGEM-T vector (Promega, Madison, USA) according to the manufacturer’s procedures used to transforms One Shot® TOP10 Chemically Competent *Escherichia coli cells* (Invitrogen, Paisley, UK). Positive transformants, based on white/blue screening, were picked up and grown at 37°C in 3 ml of liquid LB medium supplemented with ampicillin (50 μg mL^-1^). Plasmid vectors were purified using PureYield™ Plasmid Miniprep System (Promega, Madison, USA) according to the manufacturer’s procedures. Sequencing was outsourced to the BMR Genomics [47].

### Statistical analysis

Significance of differences (P < 0.05) between treatments at each time point was confirmed by *t*-test (SigmaStat for Windows Version 2.0, SPSS Inc., Chicago, IL, USA).

## Misc

Miroslav Nikolic and Stefano Cesco contributed equally to this work

## Authors’ contributions

MN, SC, ZV and RP conceived the study. MN and SC performed the nitrate transport experiments. SC, RM and MN carried out isolation of plasma membrane vesicles and performed biochemical assays. AZ, SG, and NT conducted the molecular part. MN, SC, ZV, RP, NT, AZ wrote the manuscript. All authors read and approved the manuscript for submission.
